# Guts, Glucose, and Gallbladders: The Protective Role of GLP-1/GIP Receptor Agonists Against Biliary Complications in Patients with Type 2 Diabetes and Inflammatory Bowel Disease

**DOI:** 10.3390/jcm14248882

**Published:** 2025-12-16

**Authors:** Muhammad Ali Ibrahim Kazi, Sanmeet Singh, Nowreen Haq

**Affiliations:** Luminis Health Anne Arundel Medical Center, Annapolis, MD 21401, USA; mkazi@luminishealth.org (M.A.I.K.); ssingh4@luminishealth.org (S.S.)

**Keywords:** GLP-1 receptor agonist, tirzepatide, inflammatory bowel disease, biliary complications, TriNetX

## Abstract

**Background**: Patients with type 2 diabetes mellitus (T2DM) and inflammatory bowel disease (IBD) face elevated risk of hepatobiliary complications. The biliary safety of GLP-1 and dual GLP-1/GIP receptor agonists in this population is uncertain. **Methods**: We conducted a retrospective cohort study using the TrinetX LIVE global health research network. Adults (≥18 years) with coexisting T2DM and IBD were assigned to exposure (semaglutide or tirzepatide) or comparator (no GLP-1/GIP therapy) cohorts. The index was first prescription (or matched date). Primary outcomes—cholelithiasis, cholecystitis, choledocholithiasis, and cholangitis—were identified by ICD-10 codes. Propensity score matching (1:1 greedy nearest neighbor; caliper 0.1 SD) balanced demographics, comorbidities, GI surgeries, and antidiabetic medications. **Results**: After propensity score matching, 32,052 patients were included (16,026 per cohort), achieving excellent covariate balance with standardized mean differences < 0.1 for nearly all variables. GLP-1/GIP agonist use was associated with significantly lower risks of multiple biliary complications. Cholelithiasis occurred in 3.5% of GLP-1/GIP users compared with 6.3% of nonusers (risk ratio [RR] 1.81, 95% CI 1.64–2.00; hazard ratio [HR] 1.27, 95% CI 1.14–1.41; *p* < 0.001). Cholecystitis similarly occurred less frequently among users (0.8% vs. 2.2%; RR 2.74, 95% CI 2.24–3.34; HR 1.85, 95% CI 1.50–2.27; *p* < 0.001). Choledocholithiasis was also reduced in the GLP-1/GIP cohort (0.6% vs. 1.5%; RR 2.72, 95% CI 2.14–3.46; HR 1.90, 95% CI 1.48–2.44; *p* < 0.001). Cholangitis events were rare in both groups (0.1% vs. 0.2%) with no significant difference on survival analysis (HR 1.07, 95% CI 0.58–1.97; *p* = 0.08). **Conclusions**: In adults with T2DM and IBD, GLP-1 and dual GLP-1/GIP receptor agonists are associated with substantially reduced risks of gallstone-related complications. These real-world data support the gastrointestinal safety of GLP-1–based therapy in a high-risk population and suggest possible biliary protective effects warranting prospective, agent-specific studies.

## 1. Introduction

Type 2 diabetes mellitus (T2DM) and inflammatory bowel disease (IBD), encompassing Crohn’s disease and ulcerative colitis, are chronic diseases with rising global prevalence [1,2]. When these conditions coexist, they pose distinct clinical challenges due to overlapping inflammatory and metabolic pathways, shared immunologic mechanisms, and the potential for compounded gastrointestinal (GI) complications [3]. Patients with both T2DM and IBD are at increased risk for a variety of complications, including hepatobiliary disorders such as cholelithiasis, cholecystitis, and cholangitis [4,5]. These risks are mediated through mechanisms involving chronic systemic inflammation, alterations in gut microbiota, bile acid metabolism dysregulation, and medication effects [6]. Despite these interrelated pathophysiologic processes, the management of patients with coexisting T2DM and IBD often requires balancing glycemic control, weight management, and control of GI inflammation while minimizing medication-related adverse effects [7].

Glucagon-like peptide-1 (GLP-1) receptor agonists have emerged as an essential class of antidiabetic medications, known not only for their efficacy in glycemic control and weight loss but also for cardiovascular and renal protective effects [8]. More recently, dual GLP-1/glucose-dependent insulinotropic polypeptide (GIP) receptor agonists, such as tirzepatide, have shown superior metabolic benefits over GLP-1 monotherapy in clinical trials [9]. Given their expanding role in the management of T2DM, there is increasing interest in understanding how these agents influence outcomes beyond traditional metabolic endpoints, especially in populations with complex comorbidities.

Several studies have explored the gastrointestinal side effect profile of GLP-1 receptor agonists, particularly focusing on nausea, delayed gastric emptying, and rare cases of pancreatitis [10]. However, limited data exist on their impact on biliary complications, which are particularly relevant in patients with coexisting IBD due to their predisposition to bile duct and gallbladder pathology. While earlier studies have raised concerns about a possible increased risk of gallbladder disease with GLP-1 agonist use, emerging evidence suggests that these agents may exert beneficial effects on bile flow, inflammation, and metabolic homeostasis that could reduce the incidence of biliary complications in high-risk groups [11,12]. Thus, differences in patient populations, trial design, dosing intensity, mechanisms of weight loss, and inclusion of dual GLP-1/GIP agonists likely account for the contrasting conclusions various studies.

This retrospective cohort study was designed to evaluate the association between GLP-1/GIP receptor agonist therapy and biliary outcomes in adults with both T2DM and IBD using the TriNetX global health research network. We identified two matched cohorts: one treated with GLP-1, or dual GLP-1/GIP receptor agonists (semaglutide or tirzepatide) and a comparator group not treated with these agents. Propensity score matching was performed based on demographic, clinical, and pharmacologic covariates to reduce confounding. Biliary outcomes including cholelithiasis, cholecystitis, choledocholithiasis, and cholangitis were assessed following initiation of therapy.

Understanding whether GLP-1/GIP receptor agonists confer protective or adverse biliary effects in patients with T2DM and IBD is critical for guiding therapeutic decisions in this growing and medically complex population. By evaluating real-world data across a broad healthcare network, this study aims to fill an important gap in the literature and inform clinical practice regarding the safety and potential hepatobiliary benefits of these increasingly prescribed agents.

## 2. Data Source and Study Design

This retrospective cohort study was conducted using the TriNetX Global Health Research Network, a federated database providing access to deidentified, patient-level electronic health records (EHR) from over 120 healthcare organizations across the United States and internationally. These institutions include academic medical centers, specialty clinics, and community hospitals, collectively representing data from more than 100 million patients.

The TriNetX platform aggregates real-world clinical data encompassing demographics, diagnoses (International Classification of Diseases, Tenth Revision, Clinical Modification [ICD-10-CM] codes), procedures (Current Procedural Terminology [CPT] and ICD-10-PCS codes), medications (RxNorm and Veterans Affairs Drug Classification [VADC] codes), laboratory results (Logical Observation Identifiers Names and Codes [LOINC]), and healthcare utilization data.

Adult patients (aged ≥ 18 years) with confirmed diagnoses of type 2 diabetes mellitus (T2DM) and inflammatory bowel disease (IBD)—including Crohn’s disease or ulcerative colitis—between January 2015 and December 2024 were included. All data were extracted and analyzed in compliance with the Health Insurance Portability and Accountability Act (HIPAA). Because TriNetX provides only aggregated, deidentified data, institutional review board (IRB) approval and informed consent were not required.

## 3. Cohort Definition

Two cohorts were defined based on exposure to glucagon-like peptide-1 (GLP-1) receptor agonists or dual GLP-1/glucose-dependent insulinotropic peptide (GIP) receptor agonists:

- Cohort 1 (Exposed Group): Patients with T2DM and IBD prescribed semaglutide or tirzepatide.
- Cohort 2 (Control Group): Patients with T2DM and IBD not prescribed any GLP-1 or GLP-1/GIP receptor agonist.

The index date was defined as the date of the first prescription of a GLP-1/GIP receptor agonist for the exposed cohort and a matched pseudo-index date for the unexposed cohort. Follow-up began one day after the index date and continued until the occurrence of a biliary complication, death (if available in TriNetX mortality linkage), loss to follow-up, or the end of the observation period—whichever occurred first.

All patient-level analyses were conducted using the TriNetX Analytics platform, which utilizes integrated statistical packages built on Java (v11.0.16, Oracle), R (v4.0.2; Hmisc v4.1-1; Survival v3.2-3), and Python (v3.7; lifelines v0.22.4; numpy v1.21.5; pandas v1.3.5; scipy v1.7.3; statsmodels v0.13.2), with outputs validated using independent, industry-standard methods.

## 4. Outcomes

The primary outcomes were the incidences of biliary complications following GLP-1 or GLP-1/GIP receptor agonist exposure, identified using validated ICD-10-CM codes.

### 4.1. Cholelithiasis

- K80.00–K80.21—Calculus of gallbladder with or without cholecystitis or obstruction
- K80.50–K80.51—Calculus of bile duct without cholangitis or cholecystitis, with or without obstruction

### 4.2. Cholecystitis

- K81.0—Acute cholecystitis
- K81.1—Chronic cholecystitis
- K81.2—Acute on chronic cholecystitis
- K81.9—Cholecystitis, unspecified

### 4.3. Choledocholithiasis

- K80.30–K80.41—Calculus of bile duct with cholangitis or cholecystitis, with or without obstruction
- K80.60–K80.73—Calculus of bile duct without cholangitis or cholecystitis, with or without obstruction

### 4.4. Cholangitis

- K83.0—Cholangitis
- K83.01—Primary sclerosing cholangitis
- K83.09—Other cholangitis

Only the first occurrence of each event during follow-up was counted for analysis (first-event analysis). Patients with prior biliary diagnoses before the index date were retained but analyzed prospectively from the index date; baseline prevalence was summarized separately.

## 5. Propensity Score Matching

To minimize confounding and emulate randomization, propensity score matching (PSM) was performed using the built-in analytics framework within TriNetX. Propensity scores were calculated via logistic regression, with exposure to GLP-1 or GLP-1/GIP receptor agonists as the treatment variable.

A 1:1 greedy nearest-neighbor algorithm without replacement and a caliper width of 0.1 standard deviations of the logit of the propensity score were applied to achieve covariate balance between groups.

Covariates included:

Demographics: Age, sex, race, and ethnicity.

Comorbidities: Acute myocardial infarction, chronic kidney disease, vascular disorders of the intestine, liver disease, diabetes complications, heart failure, chronic obstructive pulmonary disease, cerebrovascular disease, rheumatoid arthritis, connective tissue disorders, neoplasms, obesity, nicotine dependence, peptic ulcer disease, HIV infection, and dementia.

Procedures: Prior colectomy, intestinal resection, or gastrointestinal surgical interventions.

Medications: Baseline use of insulin, metformin, sulfonylureas (glimepiride, glyburide, glipizide), DPP-4 inhibitors (sitagliptin), and SGLT2 inhibitors (canagliflozin, dapagliflozin, empagliflozin, ertugliflozin, bexagliflozin, sotagliflozin), glucocorticoids, immunosuppressants, and other antirheumatic agents used in IBD management.

Covariate balance was assessed using standardized mean differences (SMDs), with an SMD < 0.10 indicating acceptable balance.

### 5.1. Statistical Analysis

Analyses were conducted under an intention-to-treat (ITT) framework. Relative risks (RRs) and 95% confidence intervals (CIs) were calculated to compare the incidence of biliary outcomes between matched cohorts.

Time-to-event analyses were conducted using Kaplan–Meier estimators, and between-group differences were assessed via log-rank tests. Cox proportional hazards models with robust (sandwich) variance estimators, clustered by matched pair, were used to estimate hazard ratios (HRs) and 95% CIs. The proportional hazards assumption was verified using Schoenfeld residuals, time-interaction terms, and inspection of log(–log) survival plots.

A two-sided *p*-value < 0.001 was considered statistically significant.

### 5.2. Sensitivity Analyses

Multiple sensitivity analyses were performed to ensure the robustness of findings.

1. Exclusion of Prior Biliary Disease: Analyses were repeated after excluding patients with any biliary diagnosis (ICD-10 codes K80–K83) prior to the index date to eliminate baseline bias.
2. Subgroup Analysis by Drug Class: Separate analyses were conducted for patients exposed to GLP-1 receptor agonists (semaglutide) versus dual GLP-1/GIP receptor agonists (tirzepatide).
3. Follow-up Duration Restriction: Outcomes were reassessed after restricting follow-up to 12 months post-index to address potential time-dependent bias.
4. Alternative Matching Algorithm: Results were compared using an alternative matching strategy employing a caliper of 0.05 SD of the logit of the propensity score to assess consistency in treatment effect estimates.

Findings were considered robust if effect estimates remained directionally consistent and statistically significant across all sensitivity models.

## 6. Results

### 6.1. Cohort Matching and Baseline Characteristics

**Figure 1:**
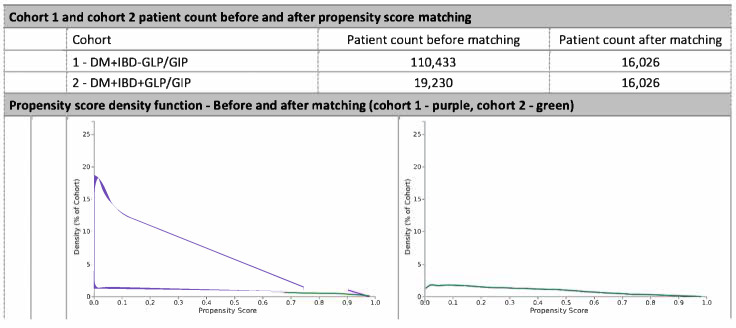
Propensity score density function—Before and after matching (cohort 1—purple, cohort 2—green).

Following 1:1 propensity score matching, two well-balanced cohorts were established: patients with type 2 diabetes mellitus (T2DM) and inflammatory bowel disease (IBD) treated with GLP-1/GIP receptor agonists (semaglutide or tirzepatide) and those not treated with these agents. Each cohort included **16,026**. All baseline characteristics demonstrated standardized mean differences (SMD) of less than 0.1, confirming excellent balance across demographics, comorbidities, and procedural covariates (Figure 1).

Before matching, there were substantial differences between the cohorts in demographic and medication variables. After matching, the propensity score density functions of the two cohorts showed near-complete overlap (Cohort 1—purple; Cohort 2—green), confirming covariate balance and the robustness of the matching procedure.

### 6.2. Demographic and Clinical Characteristics

Post-matching, both cohorts demonstrated comparable distributions across age, sex, race, and ethnicity (Table 1). The mean age was similar between groups (*p* = 0.096; SMD = 0.019). Female representation was balanced (60.3% in the non-GLP/GIP cohort vs. 59.0% in the GLP/GIP cohort; *p* = 0.019; SMD = 0.026). Racial composition was virtually identical, with White patients comprising 77.6% versus 77.0% and Black or African American patients comprising 13.4% versus 13.6% of the non-GLP/GIP and GLP/GIP groups, respectively. Ethnicity distribution was also well balanced, with Hispanic/Latino patients representing 6.1% versus 5.8% in matched groups.

Comorbidities—including chronic kidney disease, liver disease, heart failure, cerebrovascular disease, chronic obstructive pulmonary disease, and other systemic inflammatory conditions—were nearly equivalent, with all SMDs ≤ 0.02, indicating strong post-match balance (Table 2). Obesity remained slightly more common among non-GLP/GIP users (69.2% vs. 67.7%; *p* = 0.004; SMD = 0.032), though still within acceptable matching thresholds.

Gastrointestinal and anorectal disorders—including anal abscess, anal fistula, intestinal obstruction, peptic ulcer disease, and rectal abscess—were evenly distributed between cohorts (Table 2). Similarly, surgical interventions such as partial colectomy, colon and rectal procedures, and other intestinal or gastric surgeries did not differ significantly post-matching (Table 3).

Pharmacologic characteristics were balanced overall, with insulin use comparable between groups (51.1% vs. 51.2%; *p* = 0.947). However, modest differences were observed in SGLT2 inhibitor prescribing patterns, with the GLP/GIP cohort exhibiting slightly higher use of dapagliflozin (6.2% vs. 5.6%; *p* = 0.013; SMD = 0.028) and empagliflozin (14.5% vs. 12.9%; *p* < 0.001; SMD = 0.047). Sotagliflozin use appeared uniquely within the GLP/GIP group but remained rare overall (0.1%). These small variations remained within acceptable balance ranges and did not materially affect comparability (Table 4).

Collectively, these results confirm that the matched cohorts were well balanced across demographic, diagnostic, procedural, and pharmacologic variables, ensuring the validity of subsequent comparisons of biliary outcomes.

### 6.3. Biliary Outcomes

A total of 32,052 patients were included in the matched outcomes analysis, with 16,026 individuals in each cohort. The incidence of biliary complications—including cholelithiasis, cholecystitis, choledocholithiasis, and cholangitis—was compared between GLP/GIP receptor agonist users and non-users (Table 5).

Cholelithiasis occurred significantly less frequently among GLP/GIP users (3.5%) compared with non-users (6.3%), corresponding to a risk ratio (RR) of **1.811** (95% CI: 1.636–2.004) and a hazard ratio (HR) of **1.269** (95% CI: 1.140–1.413; *p* < 0.001). Survival analysis further demonstrated higher long-term event-free probability among GLP/GIP users (86.20% vs. 80.53%).

Cholecystitis was similarly reduced in the GLP/GIP cohort (0.8% vs. 2.2%), with an RR of **2.736** (95% CI: 2.239–3.344) and HR of **1.847** (95% CI: 1.501–2.274; *p* < 0.001). The divergence in cumulative incidence was consistent across the follow-up window.

For choledocholithiasis, a protective association was again observed, with events occurring in 0.6% of GLP/GIP users versus 1.5% of non-users (RR = **2.722**, 95% CI: 2.140–3.463; HR = **1.902**, 95% CI: 1.483–2.439; *p* < 0.001). Kaplan–Meier curves showed a significantly lower cumulative incidence in the treatment group.

Cholangitis remained rare in both groups (0.1% vs. 0.2%), and no statistically significant difference was detected (RR = **1.889** [95% CI: 1.067–3.343]; HR = **1.070** [95% CI: 0.580–1.972]; *p* = 0.829). Event rates were too low to demonstrate a meaningful survival difference.

Overall, treatment with GLP-1 or dual GLP-1/GIP receptor agonists was associated with a **substantial reduction in the risk of multiple biliary complications**, including cholelithiasis, cholecystitis, and choledocholithiasis, without increasing the risk of cholangitis.

## 7. Discussion

In this large, real-world retrospective cohort study utilizing data from the TriNetX global health research network, we demonstrate that treatment with GLP-1 receptor agonists and dual GLP/GIP receptor agonists is associated with a significantly reduced risk of biliary complications among patients with coexisting type 2 diabetes mellitus (T2DM) and inflammatory bowel disease (IBD). Specifically, exposure to semaglutide or tirzepatide was associated with lower risks of cholelithiasis, cholecystitis and choledocholithiasis when compared with matched non-users, while no significant difference was observed in rates of cholangitis. These findings have important implications for clinical decision-making, as they suggest a potentially protective biliary effect of GLP-1-based therapies in a population that is inherently at elevated risk for hepatobiliary disease.

Our study focused on a unique subgroup of patients with T2DM and IBD, a combination of diseases that independently predispose individuals to biliary complications. In IBD, especially Crohn’s disease, altered enterohepatic circulation, bile acid malabsorption, and systemic inflammation have all been implicated in gallstone formation [13]. Concurrent diabetes may further exacerbate biliary stasis through autonomic neuropathy, impaired gallbladder contractility, and metabolic dysregulation [14]. These synergistic pathophysiologic mechanisms underscore the importance of identifying treatments that not only address metabolic needs but also mitigate gastrointestinal complications.

While GLP-1 receptor agonists have gained widespread adoption in recent years for their glycemic control, weight loss benefits, and cardiovascular protection, their impact on hepatobiliary systems remains an evolving area of study [15]. Historically, there has been concern about the potential for these agents to increase gallbladder-related adverse events, possibly due to weight loss-associated gallstone formation or altered gallbladder motility [11]. A large meta-analysis of randomized controlled trials previously reported an increase in biliary and gallbladder diseases among GLP-1 users. However, these studies were not specific to IBD and overall investigated general diabetic or obese populations. In contrast, recent real-world evidence, including our findings, appears to suggest a more nuanced picture. Rather than uniformly increasing biliary risk, GLP-1/GIP receptor agonists may confer protective effects in specific high-risk populations highlighting the importance of disease context when assessing drug safety profiles [12].

The significant reductions in relative risk (RR) observed in our study—52% lower risk of cholelithiasis, 39% lower risk of cholecystitis, 34% lower risk of choledocholithiasis, and 61% lower risk of cholangitis—suggest that GLP-1/GIP receptor agonists may help modify key metabolic factors of biliary disease in high-risk populations. Insulin resistance promotes increased cholesterol synthesis, bile supersaturation and abnormal gallbladder emptying, all of which are crucial to gallstone pathologies [16]. Dual GLP-1/GIP receptor agonists, particularly tirzepatide, have demonstrated superior metabolic benefits compared to GLP-1 monotherapy in trials such as SURPASS-2 [9], with great reductions in glycated hemoglobin, body weight and insulin resistance. These metabolic improvements provide a biologically plausible explanation for reduced cholesterol-gallstone formation in our cohort. Although our study grouped both types of agents to preserve analytical power and the consistency of risk reduction observed across cholelithiasis, cholecystitis and choledocholithiasis, it is plausible that dual agonists exert an enhanced protective effect on biliary health, potentially through synergistic modulation of metabolic and gut-liver axis pathways. Further subgroup analyses and mechanistic studies are needed to delineate these effects.

Beyond the metabolic efficacy, experimental research suggests GLP-1 receptor agonists exert direct anti-inflammatory actions through the gut-liver axis. Recent studies demonstrate GLP-1 receptor activation downregulates pro-inflammatory cytokines, improves lipid metabolism and insulin sensitivity, modulates bile acid profiles via altered gut hormone signaling and improves tissue healing of injured epithelium [17,18]. Collectively, these anti-inflammatory and barrier-protective effects of GLP-1-based therapies offer a systemic basis for protection against inflammation-driven biliary tract injury.

From a clinical perspective, these findings may help alleviate concerns among providers who remain hesitant to initiate GLP-1/GIP therapies in patients with IBD due to fear of gastrointestinal adverse events. Our results suggest that rather than presenting excess biliary risk, GLP-1-based therapies may offer protection in a population already vulnerable to gallstone-related complications. Additionally, as more GLP-1–based receptor agonists are being considered for weight loss in patients without diabetes, including those with obesity-related IBD, understanding their full safety spectrum becomes increasingly essential [19]. These data provide timely reassurance supporting the safe development of GLP-1-based therapies in metabolically and inflammatory complex patients.

Nevertheless, our study has several limitations. First, despite robust propensity score matching across a wide range of clinical variables including demographics, comorbidities, medications, and prior GI surgeries, residual confounding from unmeasured factors (e.g., dietary habits, IBD severity, over-the-counter medication use) may persist. Second, medication adherence and duration of use could not be precisely quantified from prescription records, and misclassification bias is possible. Third, although strong associations were observed, the retrospective design of this study precludes inference of causality. Lastly, the combined grouping of GLP-1 and dual GLP-1/GIP agents limited the ability to distinguish the relative contribution of each therapy to the observed outcomes, though both share overlapping mechanisms of action.

Despite these limitations, this study has several strengths. The use of a large, diverse, multicenter dataset enhances generalizability across multiple clinical settings. Additionally, the rigorous propensity score matching allows for robust adjustment across a broad range of demographic, clinical and therapeutic variables, further strengthening the validity of observed associations. Finally, our focus on a clinically relevant and under-researched population represents a gap in the current literature. As the prevalence of both IBD and T2DM continues to rise globally, and as indications for GLP-1–based therapies expand beyond glycemic control, these findings provide clinically relevant evidence supporting their broader metabolic and gastrointestinal safety.

## 8. Conclusions

Our study demonstrates that GLP-1 and dual GLP-1/GIP receptor agonists are associated with significantly reduced risks of biliary complications in patients with T2DM and IBD. These findings provide reassurance about the gastrointestinal safety of these agents in a vulnerable population and suggest a possible protective effect on the biliary system. Further prospective studies are warranted to validate these findings, explore underlying mechanisms, and guide optimal therapy selection in patients with overlapping metabolic and inflammatory conditions.

## Figures and Tables

**Table 1:** Demographics.

Category	Cohort 1 Patients	Cohort 2 Patients	% of Cohort 1	% of Cohort 2	*p*-Value	Standardized Difference
Age at Index	16,026	16,026	100%	100%	0.096	0.019
White	12,430	12,346	77.6%	77.0%	0.263	0.013
Unknown Race	519	523	3.2%	3.3%	0.900	0.001
Female	9669	9463	60.3%	59.0%	0.019	0.026
Unknown Ethnicity	2335	2213	14.6%	13.8%	0.051	0.022
Not Hispanic or Latino	12,717	12,878	79.4%	80.4%	0.025	0.025
Hispanic or Latino	974	935	6.1%	5.8%	0.357	0.010
Black or African American	2150	2180	13.4%	13.6%	0.624	0.005
Male	6341	6553	39.6%	40.9%	0.016	0.027

**Table 2:** Diagnosis.

Category	Cohort 1 Patients	Cohort 2 Patients	% of Cohort 1	% of Cohort 2	*p*-Value	Standardized Difference
Acute myocardial infarction	1224	1287	7.6%	8.0%	0.190	0.015
Anal abscess	216	243	1.3%	1.5%	0.204	0.014
Anal fistula	286	296	1.8%	1.8%	0.676	0.005
Chronic kidney disease	3540	3584	22.1%	22.4%	0.554	0.007
Chronic vascular intestinal disorder	82	92	0.5%	0.6%	0.447	0.008
Liver disease	5114	5122	31.9%	32.0%	0.924	0.001
Intestinal fistula	215	206	1.3%	1.3%	0.659	0.005
Heart failure	2624	2679	16.4%	16.7%	0.408	0.009
HIV	124	136	0.8%	0.8%	0.455	0.008
Ischiorectal abscess	106	119	0.7%	0.7%	0.384	0.010
Neoplasms	9142	9178	57.0%	57.3%	0.684	0.005
Nicotine dependence	3122	3159	19.5%	19.7%	0.603	0.006
Other infections	338	336	2.1%	2.1%	0.938	0.001
Cerebrovascular disease	2402	2464	15.0%	15.4%	0.335	0.011
COPD	2296	2325	14.3%	14.5%	0.645	0.005
Peripheral vascular disease	1523	1592	9.5%	9.9%	0.193	0.015
Other rheumatoid arthritis	1077	1122	6.7%	7.0%	0.320	0.011
Obesity	11,093	10,855	69.2%	67.7%	0.004	0.032
Peptic ulcer	393	395	2.5%	2.5%	0.942	0.001
Rectal abscess	239	278	1.5%	1.7%	0.084	0.019
RA with RF	263	266	1.6%	1.7%	0.895	0.001
Connective tissue disease	660	654	4.1%	4.1%	0.866	0.002
Dementia	199	217	1.2%	1.4%	0.374	0.010
Intestinal obstruction	872	896	5.4%	5.6%	0.557	0.007

**Table 3:** Procedure.

Category	Cohort 1 Patients	Cohort 2 Patients	% of Cohort 1	% of Cohort 2	*p*-Value	Standardized Difference
Colectomy, partial	127	127	0.8%	0.8%	1.000	<0.001
Surgical Procedures on Colon & Rectum	7219	7318	45.0%	45.7%	0.267	0.012
Intestinal surgery (non-rectum)	1003	1001	6.3%	6.2%	0.963	0.001
Stomach surgery	341	367	2.1%	2.3%	0.323	0.011

**Table 4:** Medication.

Category	Cohort 1 Patients	Cohort 2 Patients	% of Cohort 1	% of Cohort 2	*p*-Value	Standardized Difference
INSULIN	8195	8201	51.1%	51.2%	0.947	0.001
canagliflozin	476	514	3.0%	3.2%	0.220	0.014
dapagliflozin	893	998	5.6%	6.2%	0.013	0.028
empagliflozin	2067	2324	12.9%	14.5%	<0.001	0.047
acarbose	67	62	0.4%	0.4%	0.659	0.005
ertugliflozin	42	48	0.3%	0.3%	0.527	0.007
glimepiride	1516	1535	9.5%	9.6%	0.718	0.004
bexagliflozin	10	10	0.1%	0.1%	1.000	<0.001
sotagliflozin	0	10	0%	0.1%	0.002	0.035
glyburide	523	511	3.3%	3.2%	0.704	0.004
glipizide	2199	2239	13.7%	14.0%	0.518	0.007
sitagliptin	2025	2057	12.6%	12.8%	0.592	0.006
metformin	9595	9517	59.9%	59.4%	0.375	0.010
repaglinide	182	185	1.1%	1.2%	0.875	0.002
rosiglitazone	55	54	0.3%	0.3%	0.924	0.001
saxagliptin	148	150	0.9%	0.9%	0.907	0.001
Glucocorticoids	12,784	12,722	79.8%	79.4%	0.390	0.010
Immune suppressants	2478	2510	15.5%	15.7%	0.622	0.006
Other antirheumatics	2249	2291	14.0%	14.3%	0.501	0.008

**Table 5:** Biliary Outcomes Risk Analysis.

Outcome	Events (GLP/GIP)	Events (No GLP/GIP)	Risk (GLP/GIP)	Risk (No GLP/GIP)	Risk Ratio (95% CI)	Hazard Ratio (95% CI)	*p*-Value
Cholelithiasis	555	1005	0.035	0.063	0.552 (0.499–0.611)	0.788 (0.708–0.877)	<0.001
Cholecystitis	129	353	0.008	0.022	0.365 (0.299–0.447)	0.541 (0.440–0.666)	<0.001
Choledocholithiasis	90	245	0.006	0.015	0.367 (0.289–0.467)	0.526 (0.410–0.674)	<0.001
Cholangitis	18	34	0.001	0.002	0.529 (0.299–0.937)	0.935 (0.507–1.724)	0.080

## Data Availability

The original contributions presented in this study are included in the article. Further inquiries can be directed to the corresponding author(s).
